# Reprogramming the tumor-immune landscape via nanomaterial-induced immunogenic cell death: a mini review

**DOI:** 10.3389/fbioe.2025.1635747

**Published:** 2025-07-22

**Authors:** Xiangwei Meng, Chunqing Che, Yingjie Yi, Xiaoyang Qu

**Affiliations:** ^1^ Department of Medicinal Chemistry, School of Pharmacy, Qingdao University, Qingdao, China; ^2^ Department of Drug Clinical Trials, Zibo Central Hospital Affiliated to Binzhou Medical University, Zibo, China; ^3^ Department of West Hospital Orthopaedic Trauma, Zibo Central Hospital Affiliated to Binzhou Medical University, Zibo, China; ^4^ Department of Pediatrics, Zibo Central Hospital Affiliated to Binzhou Medical University, Zibo, China; ^5^ Department of Infectious Diseases, Zibo Central Hospital Affiliated to Binzhou Medical University, Zibo, China

**Keywords:** immunogenic cell death (ICD), nanomaterials, tumor microenvironment (TME), cancer immunotherapy, targeted drug delivery

## Abstract

Nanomaterial-induced immunogenic cell death (ICD) represents a transformative approach to overcoming limitations of conventional cancer immunotherapies. Unlike traditional methods hindered by systemic toxicity and inadequate targeting, nanomaterials precisely deliver therapeutic agents and effectively modulate tumor microenvironmental factors, including hypoxia, acidity, and redox imbalance. By triggering ICD through mechanisms such as reactive oxygen species generation, tumor acidity neutralization, and hypoxia alleviation, nanomaterials facilitate potent anti-tumor immune responses, enhance dendritic cell activation, and promote cytotoxic T lymphocyte recruitment. Additionally, integrating nanomaterial-induced ICD with established immunotherapies like checkpoint inhibitors and CAR-T cells has shown promising preclinical synergy, enabling robust and lasting antitumor immunity. Despite significant translational challenges related to safety, standardization, and tumor heterogeneity, continued advances in multifunctional nanoplatform development and personalized therapeutic strategies hold substantial promise for improving cancer treatment outcomes.

## 1 Introduction

Cancer immunotherapy has emerged as a revolutionary approach in oncology, harnessing the immune system’s power to selectively identify and eliminate malignant cells (Tang et al., 2023; Liu et al., 2022). Despite significant clinical successes, a substantial number of patients exhibit limited or transient responses due to immune escape mechanisms and the immunosuppressive tumor microenvironment (TME) (Shirley et al., 2024; Czajka-Francuz et al., 2023). Immunogenic cell death (ICD) represents a critical paradigm shift within cancer therapies, characterized by the induction of regulated cell death modalities that stimulate potent anti-tumor immune responses (Galluzzi et al., 2024). Unlike conventional apoptosis, ICD involves the release or exposure of specific damage-associated molecular patterns (DAMPs), such as calreticulin (CALR), ATP, and high-mobility group box 1 (HMGB1), ultimately promoting dendritic cell activation and subsequent adaptive immunity (Arimoto et al., 2024).

Nevertheless, traditional ICD-inducing strategies, including chemotherapy, radiotherapy, and photodynamic therapy (PDT), face challenges such as nonspecific targeting, inadequate tumor penetration, systemic toxicity, and inconsistent induction of ICD biomarkers (Xie et al., 2022). Recently, nanomaterials have been explored as novel agents to address these limitations, demonstrating the capability to selectively deliver therapeutic agents, enhance ICD induction efficiency, and modulate TME conditions such as hypoxia, acidity, and redox imbalance (Ma et al., 2024). In particular, multifunctional nanoplatforms that integrate therapeutic delivery, microenvironment modulation, and immune activation hold the unique potential to both eradicate primary tumors and promote durable immune memory—an essential goal for preventing recurrence and metastasis (Wang et al., 2021). In this opinion piece, we propose that nanomaterials-driven ICD could substantially enhance cancer immunotherapy efficacy by overcoming current limitations of conventional ICD inducers, thereby transforming the therapeutic landscape. This opinion highlights how nanomaterial-induced ICD serves not only as a tumor-killing mechanism but also as a strategy for reprogramming the immunosuppressive tumor-immune landscape, ultimately enhancing the breadth and durability of antitumor immunity.

## 2 Mechanistic rationale: why nanomaterials are ideal for inducing ICD

**FIGURE 1:**
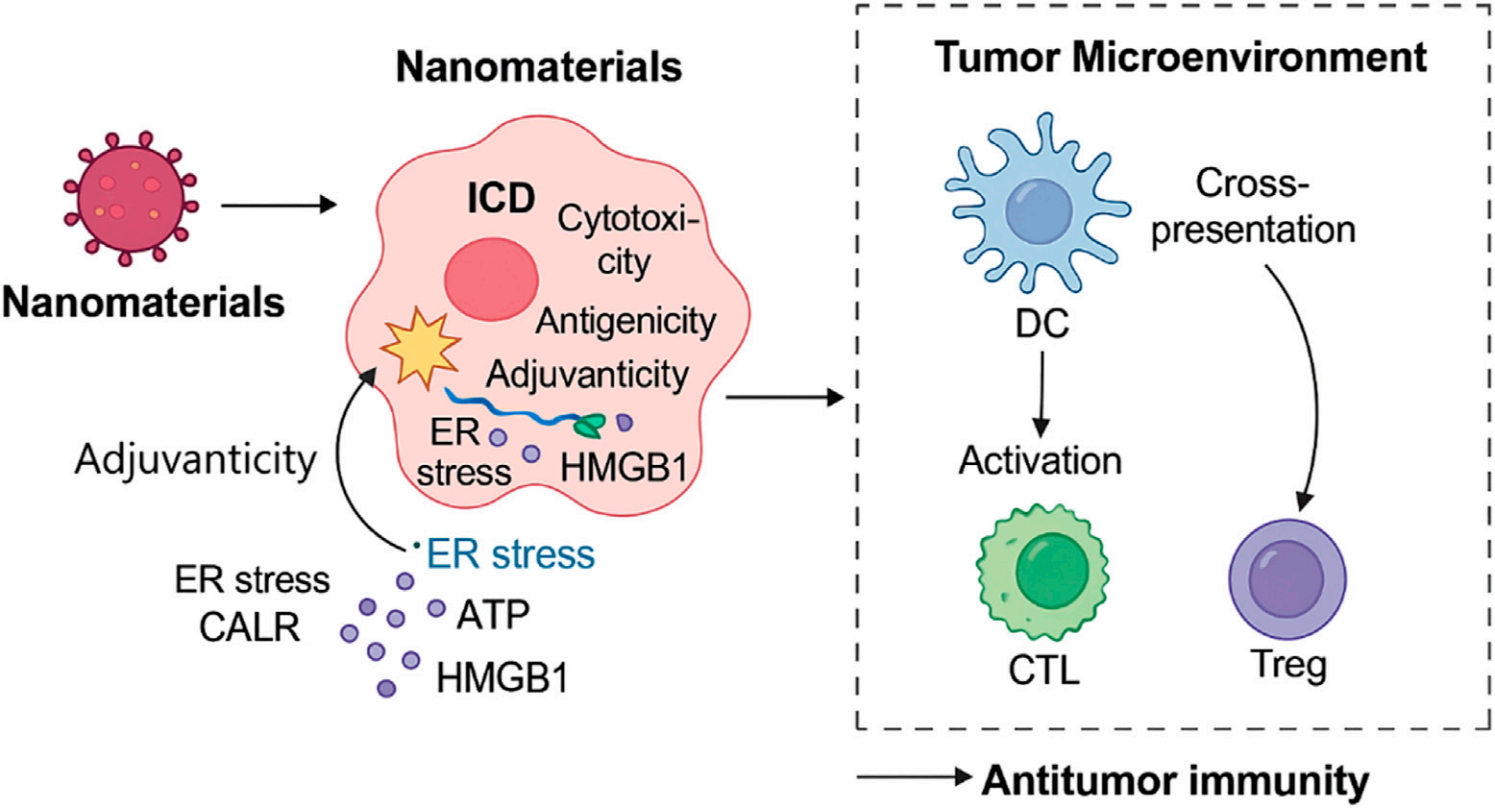
Mechanisms by which nanomaterials induce immunogenic cell death and activate antitumor immunity. This schematic illustrates how nanomaterials trigger ICD through multiple mechanisms, including ROS generation, ER stress induction, ATP and calreticulin release, and modulation of the TME. These events lead to dendritic cell activation, enhanced antigen presentation, and recruitment of CTLs, ultimately promoting a systemic antitumor immune response. The diagram also shows suppression of Tregs, further favoring immune activation. Together, these processes highlight the integrated role of nanotechnology in enhancing cancer immunotherapy via ICD pathways.

ICD efficacy fundamentally relies on four critical attributes: cytotoxicity to tumor cells, antigenicity, adjuvanticity (including endoplasmic reticulum (ER) stress and DAMPs exposure), and the permissiveness of the TME to immune cell infiltration (Dou et al., 2024; Demuynck et al., 2024). Nanomaterials uniquely address each of these components through specific physicochemical properties and functionalities. Nanomaterials can orchestrate multiple aspects of ICD through the induction of endoplasmic reticulum stress, release of DAMPs, modulation of tumor hypoxia and acidity, and enhancement of immune cell recruitment and activation (Ain, 2024). This multifunctional capability underpins their transformative potential in reshaping the tumor-immune interface for effective immunotherapy. Furthermore, nanomaterial-induced ICD facilitates the recruitment of cytotoxic T lymphocytes (CTLs), which are essential for the direct killing of tumor cells. Simultaneously, certain nanoplatforms can inhibit the activity or presence of regulatory T cells (Tregs), thereby relieving local immunosuppression within the TME and further enhancing effector T cell functions (Figure 1; Wells et al., 2024; Hiep Tran and Thu Phuong Tran, 2024). Such multifunctionality allows for synchronized tumor killing and immune priming. For example, nanoplatforms incorporating ROS generators, hypoxia modulators, and immune adjuvants enable precise spatial control of ICD and systemic immune stimulation.

Recent studies have also demonstrated that certain nanomaterials, or the therapeutic agents they deliver, can directly trigger the release of damage-associated molecular patterns (DAMPs), independent of tumor microenvironmental modulation. For instance, oxaliplatin-loaded polymeric nanoparticles induce ICD by promoting ER stress and the translocation of CALR to the cell surface (Zhao et al., 2016). Similarly, graphene oxide and zinc oxide nanoparticles have been shown to directly stimulate ATP secretion and HMGB1 release through ROS-mediated mitochondrial stress (Zhang et al., 2017). These intrinsic or cargo-mediated properties of nanomaterials highlight their multifaceted role—not only as tumor microenvironment modulators but also as direct inducers of ICD hallmarks.

Mechanistically, several representative pathways clarify how nanomaterials induce ICD. Catalytic nanozymes—such as Fe·Cu dual-atom catalysts—efficiently convert tumor H_2_O_2_ into ROS and O_2_, depleting GSH and provoking mitochondrial and ER stress, which in turn exposes CALR and releases ATP/HMGB1, driving dendritic cell maturation and antitumor immune responses (Ning et al., 2025). Additionally, the TiO_2_-based semiconductor nanosonosensitizers, under ultrasound stimulation, efficiently separate electron–hole pairs and produce ROS, inducing mitochondrial dysfunction and ICD, as demonstrated in PEG-coated TiO_2_ nanorods (Dai et al., 2017). Furthermore, Metabolism-interfering materials, such as MOF-based systems, deplete ATP and induce mitochondrial dysfunction, amplifying ICD by enhancing DAMPs exposure and improving antigen presentation—an effect corroborated by Yu et al. (2022).

Nanomaterials offer precise control over therapeutic agent delivery, significantly enhancing drug accumulation and retention within tumors. For example, lipid-based nanoparticles enhance the tumor-specific delivery and intracellular uptake of ICD-inducing chemotherapeutics such as doxorubicin and oxaliplatin, thereby promoting localized ICD without systemic toxicity (Shi et al., 2017). Additionally, engineered exosomes carrying ICD inducers like paclitaxel facilitate selective tumor cell targeting and robust ICD induction, leveraging their inherent biocompatibility and reduced immunogenicity (Chen et al., 2023). Furthermore, stimuli-responsive nanomaterials activated by tumor-specific triggers (e.g., enzymes, redox potential) enable spatiotemporal control of ICD induction, improving both safety and immunotherapeutic outcomes (Xu et al., 2024).

## 3 Representative examples and recent advances

Recent research highlights several nanomaterial strategies that effectively drive ICD, revealing great potential for enhancing cancer immunotherapy. These innovative applications are summarized through representative examples, demonstrating how nanomaterials significantly boost ICD induction by precisely modulating the TME and enhancing immune responses.

### 3.1 Hypoxia modulation

Hypoxia within tumors significantly reduces the effectiveness of various therapies, particularly radiation and PDT, both of which require sufficient oxygen to maximize cell-killing effects (Feldman, 2024). Recently, manganese dioxide (MnO_2_)-based nanoparticles have emerged as effective agents to counter tumor hypoxia. MnO_2_ catalyzes intratumoral hydrogen peroxide (H_2_O_2_) into oxygen (O_2_), directly alleviating hypoxic conditions. Enhanced oxygenation sensitizes tumors to oxygen-dependent therapies and creates a more favorable environment for ICD by facilitating immune infiltration and ROS-mediated DAMPs release. Alleviating tumor hypoxia enhances ICD primarily by restoring oxygen-dependent cellular processes critical for DAMPs generation. For instance, improved oxygenation facilitates mitochondrial respiration and reactive oxygen species (ROS) generation, both of which are essential for triggering ER stress and subsequent exposure of CALR on the tumor cell surface—an established hallmark of ICD. Moreover, increased oxygen availability amplifies the efficacy of oxygen-dependent therapies like PDT, thereby intensifying ROS-mediated cell death and promoting the release of ATP and HMGB1 from dying tumor cells (Zeng et al., 2022a; Zeng et al., 2022b). Specifically, MnO_2_ nanoparticles have shown enhanced radiation efficacy through the generation of stable DNA breaks, significantly promoting cancer cell apoptosis and subsequent ICD-associated DAMPs release (Prasad et al., 2014; Abbasi et al., 2016).

### 3.2 Tumor acidity regulation

Another critical barrier in cancer immunotherapy is tumor acidity, which contributes to immune suppression and drug resistance (Bogdanov et al., 2022). Calcium carbonate (CaCO_3_) nanoparticles effectively neutralize tumor acidity, thus significantly promoting dendritic cell (DC) activation and antigen presentation capability (Liang et al., 2023). Intratumoral injection of CaCO_3_ nanoparticles not only directly suppresses tumor growth through local pH modulation but also facilitates DC maturation and enhances cytotoxic T lymphocyte (CTL) infiltration. This combination strategy markedly potentiates immune checkpoint blockade therapies, such as anti-PD-1 therapy, through reversing tumor immunosuppression and enabling more effective antigen-specific adaptive immune responses (Shan et al., 2020).

### 3.3 Reactive oxygen species-based ICD induction

Nanomaterial-based strategies employing ROS induction have shown substantial promise in initiating potent ICD responses (Zhang et al., 2021). Nanoparticle-delivered photosensitizers precisely localize to tumor cells, generating robust and localized ROS production upon light irradiation, thus minimizing off-target toxicity and maximizing immune stimulation. Additionally, nanoparticles loaded with ferroptosis-inducing agents (e.g., RSL3) have demonstrated their ability to trigger ICD through lipid peroxidation, amplifying immune infiltration and improving anti-PD-1 treatment efficacy in preclinical models (Wang et al., 2022).

### 3.4 Synergy with immunotherapies (CAR-T and checkpoint blockades)

Recent advances have shown significant therapeutic benefits when nanomaterials-induced ICD is combined with established immunotherapeutic modalities (Demuynck et al., 2024). For instance, nanoparticles designed to alleviate tumor hypoxia or neutralize acidity have enhanced CAR-T cell therapy outcomes. Specifically, hypoxia-alleviating nanoparticles improve the persistence, functionality, and tumor-infiltrating capacity of CAR-T cells, significantly enhancing treatment efficacy in triple-negative breast cancer (TNBC) models (Dong et al., 2024). Furthermore, combining nanomaterial-driven ICD with immune checkpoint inhibitors (ICIs) has resulted in enhanced T-cell-mediated antitumor immunity, demonstrating strong therapeutic synergy and improved long-term immune memory responses (Qi et al., 2021). Similarly, osteosarcoma, a representative bone malignancy with poor immunogenicity, may benefit from nanomaterial-induced ICD strategies to overcome immune resistance and enhance responsiveness to checkpoint inhibitors or adoptive cell therapies (Hao et al., 2024). Recent evidence suggests that ICD-inducing nanomaterials, especially those coupled with adjuvants or checkpoint inhibitors, facilitate the formation of effector memory T cells and central memory T cells, providing long-term surveillance against tumor relapse. For instance, dual-delivery systems co-encapsulating oxaliplatin and CpG oligodeoxynucleotides have demonstrated robust memory T cell expansion and durable protection in murine tumor models (Deng et al., 2024).

Taken together, these representative cases underline the unique advantages of nanomaterials as versatile platforms capable of strategically modulating multiple tumor microenvironmental factors simultaneously. Such multi-modal approaches present new horizons for effective ICD induction, promising significant advancements in cancer immunotherapy.

## 4 Current challenges and limitations

Despite remarkable preclinical success, several challenges and limitations currently impede the clinical translation of nanomaterial-driven ICD strategies.

### 4.1 Safety and biocompatibility

One primary concern is the long-term safety and biocompatibility of nanomaterials. Chronic toxicity, off-target accumulation, and potential immunogenicity remain insufficiently addressed. Several nanoparticles, especially metal oxides and inorganic nanoparticles (e.g., MnO_2_, CaCO_3_), have raised concerns about potential chronic toxicity due to poor biodegradability or accumulation in vital organs (Zhang et al., 2022; Seabra et al., 2015). Thus, comprehensive long-term toxicity evaluations and strategies to improve biodegradability are essential prerequisites for clinical translation.

### 4.2 Standardization and validation of ICD induction

Another significant limitation is the absence of standardized methodologies for defining, quantifying, and validating ICD in nanomaterial-treated tumors. ICD is currently validated through variable combinations of biomarkers (CALR exposure, ATP secretion, HMGB1 release) and functional assays (vaccination assays, therapeutic immune responses). This variability complicates the comparative assessment across different nanomaterial platforms (Galluzzi et al., 2024; Galluzzi et al., 2020). Establishing consensus guidelines and standardized preclinical models to consistently assess ICD biomarkers and immune responses is urgently needed to accelerate translation into clinical practice.

### 4.3 Tumor heterogeneity and immune escape

Tumor heterogeneity remains a major obstacle, limiting the efficacy of ICD-based therapies. Intratumoral heterogeneity significantly influences responses to therapies, including ICD induction, due to variable expression of ICD markers, metabolic adaptations, and immune evasion mechanisms (Yan et al., 2025). Furthermore, tumors may develop resistance through upregulating immunosuppressive pathways (e.g., IDO1, PD-L1) or impairing antigen processing and presentation machinery. Strategies to overcome tumor heterogeneity and suppress immune escape require deeper mechanistic understanding and adaptive combination therapies.

## 5 Perspectives and future directions

Looking ahead, nanomaterial-mediated ICD induction holds significant potential for transformative clinical applications, driven by multidisciplinary advances in nanotechnology, immunology, and oncology.

### 5.1 Integrated multifunctional nanoplatforms

Future research should emphasize the development of multifunctional nanoplatforms capable of simultaneously addressing multiple barriers within the TME. Integrated platforms capable of concurrent oxygen generation, acidity neutralization, redox modulation, and controlled drug release will provide synergistic enhancements in ICD induction and immune activation (Zhu et al., 2023; Luo et al., 2025). Emerging “smart” nanoplatforms, which can respond dynamically to tumor-specific triggers (e.g., low pH, hypoxia, enzyme overexpression), represent promising next-generation approaches.

### 5.2 Precision nanomaterials for specific cell death pathways

Another promising direction involves precision nanomaterials engineered to selectively trigger specific cell death pathways such as ferroptosis, pyroptosis, or cuproptosis. Such selective cell death induction could enhance immunogenicity while minimizing off-target damage (Sahoo and Manna, 2025). For instance, ferroptosis-targeting nanoparticles can amplify ICD through selective lipid peroxidation, while pyroptosis-inducing platforms (e.g., gasdermin activators) can drive robust inflammasome activation and potent antitumor immunity (Wang et al., 2025).

### 5.3 Personalized and combinatorial immunotherapies

Personalized and combinatorial strategies integrating ICD-inducing nanomaterials with established immunotherapies (e.g., ICIs, CAR-T, cancer vaccines) hold significant translational promise. Personalized ICD vaccines created from tumor-derived neoantigens combined with nanomaterials can achieve highly specific and effective antitumor responses (Yang et al., 2024). Moreover, the co-delivery of ICD inducers with checkpoint inhibitors and engineered immune cells may dramatically enhance therapeutic efficacy, immune memory, and clinical outcomes.

## 6 Concluding remarks

Nanomaterials-driven ICD represents a paradigm shift in cancer immunotherapy, uniquely capable of orchestrating powerful antitumor immunity by strategically modulating the tumor immune microenvironment. Although preclinical studies have provided robust evidence for their therapeutic potential, significant challenges such as safety, standardization, and tumor heterogeneity remain to be overcome.

To fully realize the clinical promise of nanomaterial-mediated ICD, a concerted effort is required from multidisciplinary research teams across bioengineering, immunology, oncology, and clinical medicine. Rigorous mechanistic research, standardized validation frameworks, and adaptive clinical strategies will be critical in translating nanomaterial-driven ICD into effective, personalized cancer treatments. Ultimately, continued innovation in this exciting field could transform how clinicians harness the immune system to fight cancer, providing powerful new tools to improve patient outcomes and survival.
